# Advanced optical assessment and modeling of extrusion bioprinting

**DOI:** 10.1038/s41598-024-64039-y

**Published:** 2024-06-17

**Authors:** Zan Lamberger, Dirk W. Schubert, Margitta Buechner, Nathaly Chicaiza Cabezas, Stefan Schrüfer, Nicoletta Murenu, Natascha Schaefer, Gregor Lang

**Affiliations:** 1 Department for Functional Materials in Medicine and Dentistry, University Hospital of Würzburg, 97070 Würzburg, Germany; 2 Department of Materials Science and Engineering, University of Erlangen-Nuremberg, 91058 Erlangen, Germany; 3 Institute for Clinical Neurobiology, University Hospital of Würzburg, 97078 Würzburg, Germany

**Keywords:** Bioprinting, Biofabrication, Bioink, Extrusion, Modeling, Rheology, Biophysics, Implants, Biomedical engineering, Gels and hydrogels, Rheology

## Abstract

In the context of tissue engineering, biofabrication techniques are employed to process cells in hydrogel-based matrices, known as bioinks, into complex 3D structures. The aim is the production of functional tissue models or even entire organs. The regenerative production of biological tissues adheres to a multitude of criteria that ultimately determine the maturation of a functional tissue. These criteria are of biological nature, such as the biomimetic spatial positioning of different cell types within a physiologically and mechanically suitable matrix, which enables tissue maturation. Furthermore, the processing, a combination of technical procedures and biological materials, has proven highly challenging since cells are sensitive to stress, for example from shear and tensile forces, which may affect their vitality. On the other hand, high resolutions are pursued to create optimal conditions for subsequent tissue maturation. From an analytical perspective, it is prudent to first investigate the printing behavior of bioinks before undertaking complex biological tests. According to our findings, conventional shear rheological tests are insufficient to fully characterize the printing behavior of a bioink. For this reason, we have developed optical methods that, complementarily to the already developed tests, allow for quantification of printing quality and further viscoelastic modeling of bioinks.

## Introduction

The aim of biofabrication in the context of tissue engineering and regenerative medicine is to produce living, bio-functional tissue. This can be used, for instance, as tissue models for testing medical drugs or for tissue replacement and tissue reconstruction in cases of organ damage^1^. To achieve this, three-dimensional structures with highly defined, spatially organized properties made from cell-containing biomaterials need to be created^2^. This requires bottom-up processes, which are currently implemented using 3D printing techniques. The methods include, among others, stereolithography, inkjet, laser-assisted, and micro-extrusion fabrication. Fused filament printing is often chosen due to its simplicity, flexibility, and high material throughput^3,4^. The materials used in this process are cell-laden hydrogels or their precursors, which are directly linked after extrusion through various possible mechanisms such as ions, light, or chemical crosslinking agents to form stable networks^5^. The core criteria for producing functional tissue models conducting bioprinting can be simplified into four phases:

- *During printing*: Cell survival must be ensured. Critical factors include shear and tensile forces in the nozzle, and the key-parameters are the rheology of the bio-ink and the nozzle's geometry.
- *Immediately after printing:* The scaffold's shape fidelity must be ensured, the print resolution must be sufficient to allow diffusion of nutrients and oxygen to the cells inside and prevent hypoxia. Critical factors are crosslinking kinetics of the polymer network and porosity of the hydrogel.
- *Mid-term:* The hydrogel must mechanically match the physiological requirements of the target tissue, and cell proliferation and migration must be ensured. Critical factors are structural composition of the fibrillar network, cell density, and organization.
- *Long-term:* Tissue maturation must occur. Critical factors are cell mobility and performance, control of tissue maturation by signaling molecules, biodegradation of the artificial matrix, and substitution by the natural extracellular matrix.

**Figure 1:**
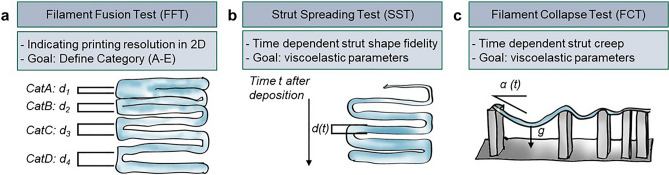
Schematic overview on selection of different experimental print tests.

Thus, the initial steps in analyzing new bio-inks involve biological testing (biocompatibility of the matrix) on one hand, and printing trials on the other, to evaluate the ink's suitability for processing into a three-dimensional construct. Both factors are equally crucial and act as knockout criteria for the respective system. Since cell biological testing is time consuming and associated with high costs for cell culture, as well as for the required agents like staining kits, it is advisable to first check the printability and define the process-related conditions of the bio-ink system—for instance, the concentration-dependent printing behavior of the ink and the respective required printing parameters. Some essential information about the printability of a hydrogel can already be obtained from shear rheological tests^6^. Schwab et al. mention parameters like flow behavior, yield stress, elastic recovery, shear stress, and damping factor tanδ^7^. The shear-thinning behavior of a bio-ink, for example, affects not only the required pressure but also the internal shear forces to which the cells are exposed. Elastic recovery provides insight into the time-dependent response of the material after shear-induced deformation and, combined with yield stress and the damping factor, plays a crucial role in shape stability. Meanwhile, shear stress influences cell behavior and print resolution. Nonetheless, additional printing trials are necessary to test and possibly optimize a bio-ink for biofabrication. To carry this out systematically, quality indicators must be established to quantitatively categorize the printing results. It must be considered that there are many different types of bio-inks, and as many of them as possible should be analytically captured^5,8^. Moreover, even within extrusion printing, there are various methods like Embedded Bioprinting, Co-axial Bioprinting, Multi-nozzle Multi-material Bioprinting, Single-nozzle Multi-material Bioprinting, etc^9,10^. In addition to categorizing the inks with their respective process, such tests should also provide insights into achievable scaffold designs and assist in optimizing, for example, G-codes for printing with a specific ink^8^. In the past, several coherent tests have been established in this regard^2,6,9^. Some frequently applied tests are schematically illustrated in Fig. 1

In bioprinting assessments, several tests gauge the behavior of printed structures. The Filament Fusion Test (FFT) evaluates the thickness of a printed strand by employing a narrowing pattern of printed lines. As the spacing between the lines decreases, it eventually leads to the fusion of two neighboring strands^11–14^. Another version involves the printing of a rectangular pore to determine material spreading, comparing the theoretical to the actual perimeter area within the pore. Since these tests are not typically recorded in a time-dependent manner, they seldom provide insights into the viscoelastic properties of the material^12,15–20^. The Filament Collapse Test (FCT) utilizes a substrate with pillars spaced at varying gap lengths. A strand is printed to bridge these gaps. Following this, the Collapse Area Factor (C_f_) is computed, which signifies the percentage of the actual area (after the suspended filament deflects) relative to its theoretical value^13,15,21^. Other research has focused on the deflection angle of the filament across the half-gap distance L as an indicator for strand stability^12^. Moreover the data were correlated with a simple physical model based on the equilibrium of gravitational forces and tension forces in the material. However, this model does not account for the elongation and consequent reduction in the cross-sectional area of the strand^22^. Evidence also supports the applicability of these tests to microgel-based bioinks, such as alginate (Alg), gelatin methacrylate (GelMA), and Alg–GelMA microgels^20^. Although not quantified beyond the critical gap distance, it's essential to note the time dependency. For instance, a strand was observed to remain stable for a few minutes, but sagged significantly after extended time succumbing to gravitational forces^17^. In other research, the deflection angle was measured 20 s post-deposition, though deformation continued at a slower pace, as discerned from a 24-h creep analysis of the hydrogels^22^. Elsewhere, the filament was captured immediately post-deposition in a FFT, to prevent undesired material spreading^14^. Given the diverse methods of conducting similar experiments, it is essential to emphasize the unification of measurement procedures to enhance comparability, especially when incorporating time dependency into such evaluations^23^. Moreover, it's noteworthy that the FCT strand stability isn't merely material-dependent. Aspects like the nozzle diameter and filament thickness play a pivotal role, as illustrated recently by Ravoor et al.^18^. On the other hand, the nozzle dimensions and the applied pressure have a significant impact on cell survival, as recently shown in a study by Han et al. They managed to establish an experimentally verified model to quantitatively describe the ratio of damaged cells^24^. To more clearly highlight the impact of material mechanics, additional standardized classic mechanical tests, such as tensile testing can be conducted also with hydrogels^25^. It has been highlighted that the extrusion process significantly alters the mechanical properties of printed hydrogel constructs, affecting both compressive and tensile stresses. These parameters are vital not only for the scaffold's overall stability but also because they mimic essential factors that influence cell behavior^26^.

Viscoelastic modeling that links rheological assessment to printing performance can serve as a powerful tool for systematically describing the time-dependent behavior of materials during flow and at rest. For instance, theoretical and experimental evidence has revealed a correlation between the spreading behavior of hydrogels and the η_1_ parameter of the augmented Burgers model^19,27,28^. Such methodologies pave the way for simulation approaches, and as the field of 3D bioprinting advances, there is an increasing demand for simulations to predict the printability of bioinks and their subsequent effects on cells. In a recent study by Göhl et al., the evaluation of rheology and its modeling, utilizing the Linear form of the Phan–Thien and Tanner (PTT) model, combined with the surface tension model and the Quasi-dynamic contact angle model, enabled the implementation of print simulations^29^. These simulations accurately represented the dispensing of inks during 3D bioprinting. Computational modeling will progressively assume a pivotal role in the appraisal of bioinks^5^. This was also demonstrated in the context of machine learning applying Bayesian optimization, a sample-efficient optimization algorithm, which was successfully integrated into an experimental bioprinting process^30^. The objective was to pinpoint the optimal printing parameters. Compared to the traditional trial-and-error optimization approach, which can be labor-intensive and time-consuming, machine learning methods offer a potent tool for enhancing the efficiency of parameterizing the bioprinting process.

In summary, it can be said that essentially all the necessary tools for the systematic analysis, modeling, and simulation of bio-inks are already available. However, to fully unleash this potential, datasets of different inks processed with various devices under different conditions are required. These datasets must be as reproducible and comparable as possible. This is already largely implemented in shear rheology. However, there is still a significant need for standardization and optimization in printing tests. To obtain comparable data regardless of the 3D printer, we developed a simple setup that allows for reproducible imaging of printing tests, introduced in “A modular setup for standardized imaging during print test procedures” section. The printing process is recorded from below (FFT, SST) or from the side (FCT). A patterned plate in the background enables the documentation of additional optical information (refraction). This forms the basis for applying optical data assessment of time-dependent strut spreading using physical models. “Optical data assessment: Strut-spreading” section introduces the optical data assessment of strut-spreading, including the theoretical model and its practical application, particularly with Alginate-based bioinks. Additionally, imaging from the side provides access to strut-trajectory data, which can be modeled to investigate the elongational viscosity of bioinks during printing, as shown in “Optical data assessment: Strut-trajectory” section. Furthermore, since cell-loading of bioinks might impact rheology and printability, in “Impact of cell loading” section, we investigated the impact of cell loading densities representative of biofabrication approaches. In combination, the test setup, along with the evaluation protocol, can make a valuable contribution to the characterization of bio-inks. The data obtained are suitable for future computational analytics approaches. They complement shear rheological tests, which primarily provide insight into the shear forces to which cells are exposed in the nozzle during the printing, and mechanical tests of the static constructs, which represent the long-term stability and mechanical environment of the embedded cells. The optical approach is therefore used for documentation and quantitative evaluation of the extrusion behavior and the associated viscoelastic effects immediately after printing.

## Results

### A modular setup for standardized imaging during print test procedures.

**Figure 2:**
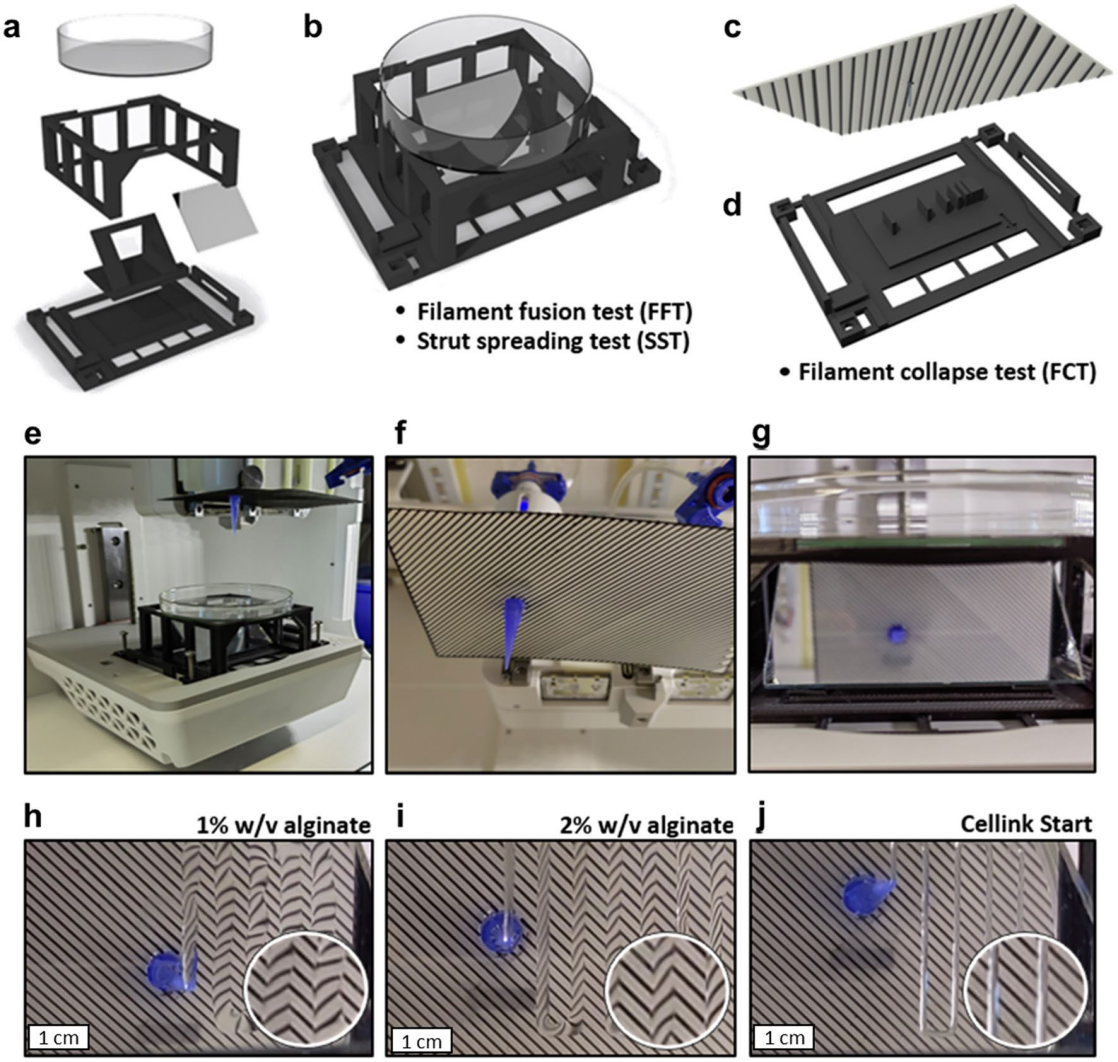
Components of the Printbase setup: An exploded view (**a**) of the components used for the FFT and SST test setups (**b**). To obtain additional information and improved resolution of the strut geometry, a patterned plate is mounted near the nozzle, acting as a background for imaging (**c**). Pillar setup for the FCT test (**d**). Print setup for the SST with the attached patterned plate for advanced imaging (**e**), view from below of the printing nozzle and the mounted plate with the pattern (**f**), view of the mirror with the printing platform in the foreground and the nozzle and patterned plate in the background (**g**). Example for SST-imaging with the patterned plate in the background (**h**–**j**). The geometry and thus the dimensional stability of the strand can be calculated from the refraction of light at the printed strand. This novel evaluation strategy and set-up is patented under the reference number DE102023206928.8^31^.

To obtain optical data from real printing experiments, a novel setup has been developed, enabling the effortless generation of imaging data from below the print plane. As depicted in Fig. 2, this setup comprises a platform with a base area matching the geometric dimensions of a well-plate, allowing it to be inserted seamlessly into any commercially available bio-printer. Furthermore, various elements can be affixed onto this platform. A configuration suited for filament fusion tests and strut-spreading tests is presented in Fig. 2b. Herein, imaging is achieved by filming through a mirror positioned to capture through the transparent print substrate, thereby generating high-quality in-situ recordings of filament behavior. This approach offers the advantage of observing temporal effects without necessitating the transportation or manipulation of printed constructs to a microscope. Additionally, imaging enhancements can be achieved by utilizing a specially patterned background, exemplified in Fig. 2c, enabling clear resolution of previously difficult to visualize inks (Fig. S1a–c). Figure 2e–g show the real installation of the modular setup in a bioprinter, which was used to image the strut spreading of different Bioinks (Fig. 2h–j). This optical imaging method simply reveals the cross-sectional area of the strut deposited on the transparent substrate. By combining the cross-sectional area with the known print speed, the throughput Q can be calculated. This calculation offers the possibility of implementing closed-loop control in a bio-printer, detailed in a patent and implemented by RevoBITs under the acronym ASTRID (Advanced STrut Investigation Device)^31^. Moreover, the setup can be applied to conduct filament collapse tests by simply attaching the insert illustrated in Fig. 2d.

**Figure 3:**
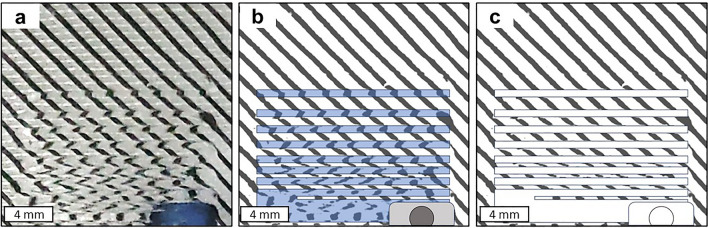
Original photo of an in-gel printed 5% GelMA bioink performing a strut fusion test (**a**). Image processing techniques can be employed to distinctly identify the strut, as the edges become discernible due to optical refraction (**b**). Ultimately, this allows the creation of a precisely defined mask of the printed strut (**c**), facilitating the assessment of printing quality.

As demonstrated in Fig. 3, the advantages of the optical setup can also be extended to in-gel printing. Conventionally, visualization of the printed strut is often problematic, especially when combining a transparent ink with a transparent support bath, rendering it practically invisible. While this is sometimes solved by the addition of dyes, these nonetheless, depending on concentration, influence ink composition and light crosslinking results. Conversely, printing into the support bath while using the striped background allows even inks that were otherwise practically invisible to be visualized without any further additives. As shown with the examples of a 5% GelMA ink in a 1% Xanthan gum/0.2% hyaluronic acid support bath, the difference in light refractive properties of the support bath/ink interface was sufficient to visualize the strut and easily quantify it (Fig. 3b,c), as opposed to attempts using a regular microscope (Fig. S1d and e). This renders this method extremely useful for the evaluation of in-gel printing quality using the same setup as for regular bioprinting evaluation without the need for further modification.

Combined with optical analysis programs and machine learning techniques, this reliable visualization method may in the future enable more reliable automated calibration of printing parameters and improve quality control mechanisms for extrusion-based printing approaches.

### Optical data assessment: Strut-spreading

#### Theoretical model

**Figure 4:**
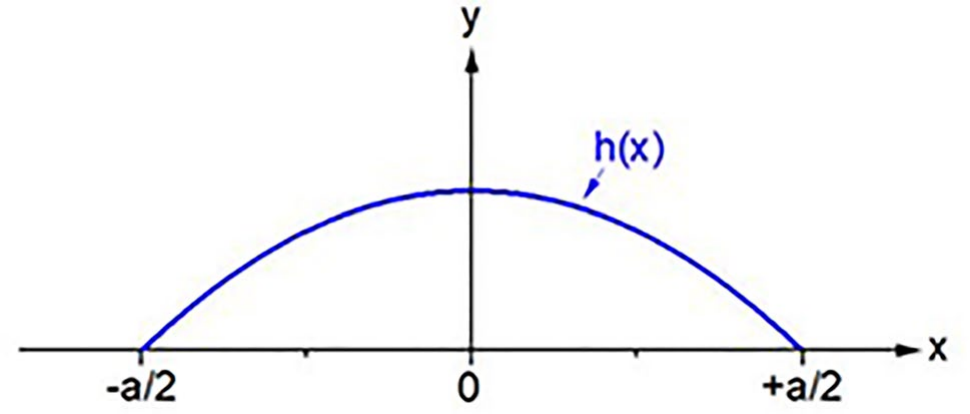
Parabolic strut shape *h(x)* where “*a*” is the width of the strut.

As demonstrated in Fig. 2h–j and Fig. 3 it's apparent that the diagonal lines within the pattern undergo a virtual rotation due to the strut. In this context, the strut functions as a cylindrical lens as shown in Fig. 4, and the optical rotation can be effectively explained using matrix formalism^31^.

This optical analysis yields a significant outcome: the curvature at the vertex ρ of the strut becomes accessible. Recent theoretical work indicates that the shape of the strut can be defined by a parabolic function^27^:

1 $$h\left(x\right)=H\cdot \left(1-{\left(\frac{2x}{a}\right)}^{2}\right)$$

The curvature at the vertex can be readily determined by taking the second derivative of Eq. (1):

2 $$\rho =\frac{8H}{{a}^{2}}$$

Therefore, the diagonal pattern allows for the direct determination of the two pertinent parameters: "*a*," representing the strut width, and "*H*," signifying the height of the strut at the vertex. Consequently, the optical analysis provides the function *h(x)*, and calculating the cross-sectional area (κ) of the strut follows simply by:

3 $$\kappa ={\int }_{-\frac{a}{2}}^\frac{a}{2}h\left(x\right)\cdot dx=\frac{2}{3}\cdot H\cdot a$$

This procedure can be further employed to establish a closed-loop control mechanism for regulating the throughput (*Q*) in bio-printing, where a specific print speed ${v}_{p}$ is predefined^31^:

4 $$Q= \kappa \cdot {v}_{p}$$

Thus, in the case of transparent bio-inks, the method offers an attractive alternative to conventional practices such as weighing the construct or measuring the printed volume based on the level of the bio-ink reservoir in the printer. The latter two methods are typically offline measurements and do not provide insights into the stability of the printing process—whether a continuous and stable volume flow can be achieved. However, this information can also be determined with non-transparent inks, especially since the background pattern can be used to automatically read the contours of the deposited strand in real-time, serving as an additional indicator of print quality.

#### Practical example: alginate-based bioinks

**Figure 5:**
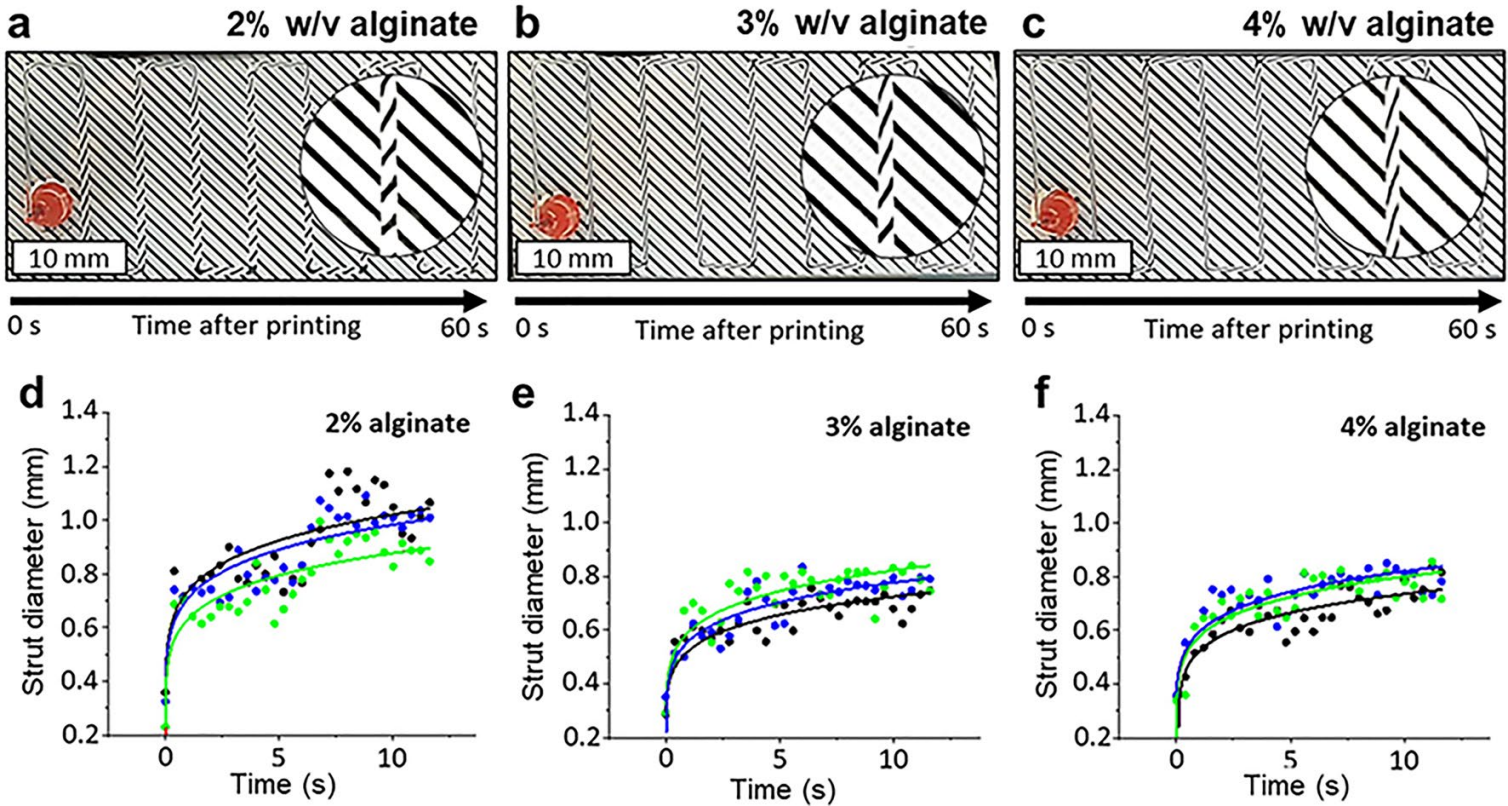
A simple meander pattern was printed using 2, 3 and 4% (w/v) alginate solutions. Images were recorded right at the end of the print (**a**–**c**). Spreading kinetics of different alginate concentrations were obtained through the analysis of the images (**d**–**f**). The three different colors correspond to three distinct experiments. The solid lines represent fits based on the theoretical model in Eq. (6).

To demonstrate the reliability of evaluating the printing results by combining the new optical setup with the models presented here, a series of measurements were conducted using various alginate concentrations (2%, 3%, and 4%). For this purpose, a simple meander pattern was printed, and the printing duration consistently lasted 60 s. The process was recorded from below using the new setup, and a screenshot was captured at the moment when the printing was completed. Consequently, the strand is freshly extruded at the end of the nozzle, while at the other end, 60 s have already elapsed. By examining the intervals between these steps, the strand could be observed over time, and the spreading of the strand was analyzed. All printing experiments were conducted under uniform conditions with a consistent mass flow of ca. 0.25 mg/s and a printing speed of 5 mm/s. Consequently, it is apparent that when the throughputs are set equal across all experiments, a comparative analysis of spreading kinetics for the three concentrations was possible. Figures 5a–c and S2a–c vividly illustrates how the spreading over time alters the curvature of the strut at the vertex, systematically rotating the line pattern. Without delving into specifics, the calculated cross-sectional areas along the strut, utilizing the aforementioned approach, remain relatively constant within experimental error. This constancy persists due to negligible evaporation over the investigated time frame of 12 s. Analyzing the spread over time in incremental steps of 2 mm along the strut's width, the experimental data were fitted using the equation derived from the recent theory^27^.

5 $$a\left(t\right)={\left(\frac{{\gamma }_{L}\cdot 18\cdot 7\cdot {\kappa }^{3}}{\lambda \cdot \eta }(t+{t}_{0})\right)}^{1/7}$$

where ${\gamma }_{L}$ is the surface tension and $\eta$ the viscosity of the ink. The already introduced cross section area $\kappa$ of the strut is constant and $\lambda$ a universal constant and ${t}_{0}$ is an experimental delay time therefore the equation can be simplified having only two adjustable parameters, K and ${t}_{0}$.

6 $$a\left(t\right)=K\cdot {\left(\frac{t+{t}_{0}}{s}\right)}^{1/7} \quad (\text{The "s" in the denominator means second})$$

It is apparent from Fig. 5d–f that Eq. (6) provides a good fit to the data, thereby experimentally validating the recent model^27^. The K values obtained from the fitted curves are (692 ± 54) 10^–3^ mm (2%w/v), (558 ± 35)⋅10^–3^ mm (3%w/v), and (566 ± 31) 10^–3^ mm (4%w/v). Analysis from Table S1 indicates that the delay time, t_0_, consistently approaches zero, and the fit error attributed to the data fitting exceeds the value itself. Hence, it is reasonable to assume t_0_ = 0 based on these observations.

Considering that the alginate is primarily submerged within water at low concentrations, the surface tension can be approximately considered constant for the three investigated alginate concentrations. Therefore, the following relationship should hold:

7 $$K\sim {\eta }^{-1/7}$$

with a typical concentration dependency of the polymer solution (here water and alginate) in the low concentration regime^32^.

8 $$\eta (c)= {\eta }_{S}\cdot {\left(1+\frac{\left[\eta \right]\cdot c}{3}\right)}^{3}$$

However, it is expected that the K value would decrease with increasing concentration. Surprisingly, this trend is observed only from 2 to 3%w/v. An explanation for the result observed at 4%w/v is that the surface tension also increases with concentration, thereby compensating for the significant increase in viscosity. To verify the applicability of the method for a commonly used bioink in biofabrication, a comparative measurement with 5% w/v GelMa was conducted (Fig. S3). It was found that the K-value of 5% w/v GelMa was 504 µm ± 50 µm (n = 3), which is of the same order of magnitude as 3% w/v alginate (K-value: 558 µm ± 35 µm). Thus, it could be demonstrated that the method is also suitable for quantitatively comparing different bioink systems.

### Optical data assessment: Strut-trajectory

#### Theoretical model

**Figure 6:**
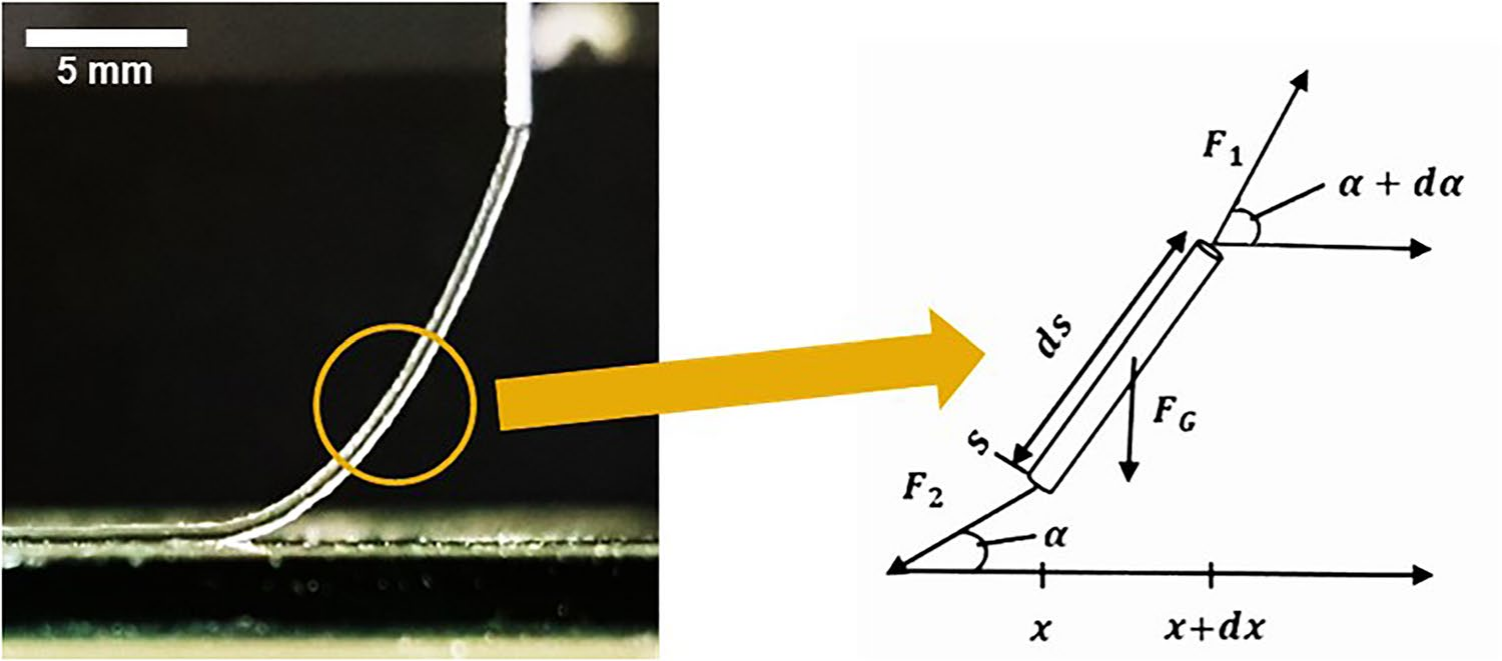
Image of alginate strut during printing (left) and theoretical interpretation thereof (right).

As mentioned before, elongational flow during bioprinting can play a crucial role, not only in the context of printing results, but also with respect to cell survival. Thus, another method of analyzing optical data to gain a deeper understanding of the interplay between material and process is based on the behavior of the strand during extrusion^33^. In this context, the viscoelastic properties of the bio-inks play an essential role. When considering the shape of a freely hanging strand during the printing process, its trajectory can be divided into many infinitesimally small cylindrical elements of length ds (Fig. 6).

Here, s represents the coordinate along the strut, *α* denotes the angle against the x-axis at position *x*, and *α* + *dα* at position *x* + *dx*. Three force contributions come into play: *F*_*1*_ and *F*_*2*_ due to elongation at positions *x* and *x* + *dx*, respectively, and *Fg* due to gravity:

9 $${F}_{1}={\eta }_{E}\cdot \dot{\varepsilon }(s+ds)\cdot A(s+ds)$$

10 $${F}_{2}={\eta }_{E}\cdot \dot{\varepsilon }(s)\cdot A(s)$$

$\dot{\varepsilon }$ is the elongation rate, ${\eta }_{E}$ the elongation viscosity and A the cross-section area.

11 $${F}_{G}=dm\cdot g=\rho \cdot g\cdot dV=\rho \cdot g\cdot A(s)\cdot ds$$

Force balance in x-direction yields:

12 $${F}_{2}\cdot \text{cos}\left(\alpha \right)={F}_{1}\cdot \text{cos}(\alpha +d\alpha )$$

Force balance in y-direction yields:

13 $${F}_{2}\cdot \text{sin}\left(\alpha \right)+\rho \cdot g\cdot A\left(s\right)\cdot ds={F}_{1}\cdot \text{sin}(\alpha +d\alpha )$$

Dividing Eq. (13) by (12) yields with

14 $$\dot{\varepsilon }=\frac{dv(s)}{ds}$$

15 $${\eta }_{E}=\frac{\rho \cdot g}{\frac{d\alpha (s)}{ds}\cdot \frac{dv(s)}{ds}}\cdot \text{cos}(\alpha \left(s\right))$$

or with

16 $$\text{cos}\alpha =\frac{dx}{ds}$$

17 $${\eta }_{E}=\frac{\rho \cdot g}{\frac{d\alpha (x)}{dx}\cdot \frac{dv(s)}{ds}}$$

Considering additionally the surface tension $\gamma$, Eqs. (15) and (17) can be augmented to:

18 $${\eta }_{E}=\frac{\rho \cdot g}{\frac{d\alpha (s)}{ds}\cdot \frac{dv(s)}{ds}}\cdot \text{cos}\left(\alpha \left(s\right)\right)-\frac{2\cdot \gamma }{d\left(s\right)\cdot \frac{dv\left(s\right)}{ds}}$$

19 $${\eta }_{E}=\left(\frac{\rho \cdot g}{\frac{d\alpha (x)}{dx}}-\frac{2\cdot \gamma }{d\left(s\right)}\right)\frac{1}{\frac{dv(s)}{ds}}$$

From Eqs. (18) and (19) it is evident that a detailed analysis of the strut, trajectory and diameter d enables access to the elongation viscosity when considering continuity of the volume:

20 $$v\left(s\right)=\frac{Q}{A(s)}=\frac{4\cdot Q}{\pi \cdot {d(s)}^{2}}$$

where Q is the throughput set by the bioprinter.

It's important to note that although elongation viscosity is typically time-dependent, experimental manipulation allows for the independent variation of throughputs, print speeds, elongation rates, and time (post-nozzle passage). The aforementioned equations and a specifically designed setup are detailed in a recent patent known as BERIT (Best Elongation Rheology Investigative Testing)^33^, developed by the company RevoBITs. An experimental demonstration is provided in the following experimental section. However, it's important to note that the model described above represents the simplest case and therefore provides only a rough approximation of reality. Future considerations should involve more comprehensive models.

#### Practical example: alginate-based bioinks

**Figure 7:**
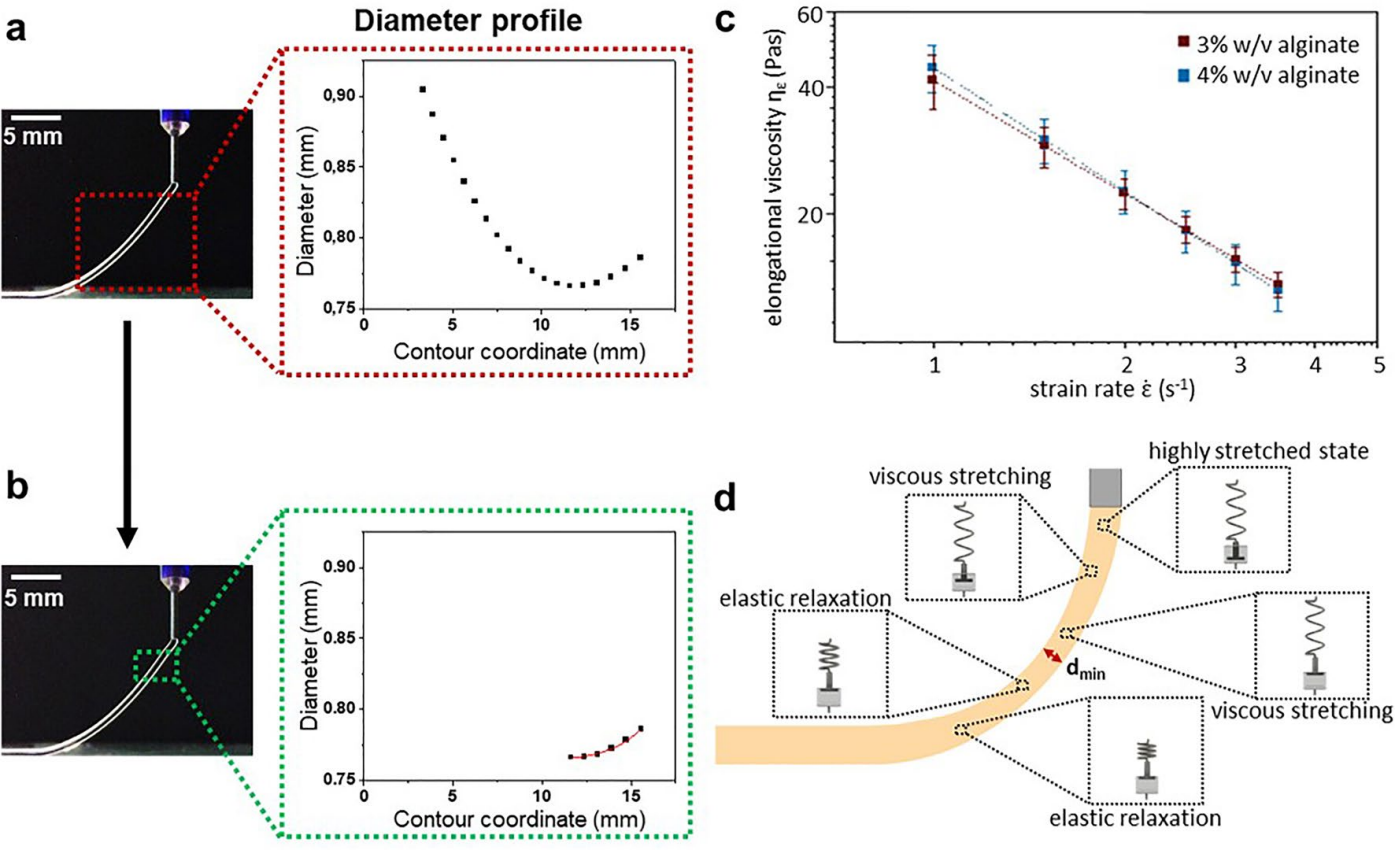
Parabolic-like shaped diameter profile of the printed strand of alginate solutions (**a**), with further evaluation of the profile near the nozzle (**b**). The elongational viscosity of 3% and 4% w/v alginate, calculated using Eq. (19), is depicted as a function of the strain rate (**c**). Additionally, the hypothesis regarding the diameter profile (**a**), as explained in the text, is presented in (**d**).

To verify the model, alginate solutions (3%w/v and 4%w/v) were printed, and their extrusion behavior was evaluated based on the strut trajectory. As illustrated in Fig. 7a and b, the diameter profiles of alginate solutions exhibit a parabolic-like shape, indicating two distinct regions: a viscous stretching phase followed by elastic strut recovery. These phenomena are further elucidated in Fig. 7d using a simplified spring-dashpot Maxwell model and suggesting a sequence of events: initially, a highly elastic stretched ink exits the nozzle, followed by viscous stretching, which reduces the strut diameter. Subsequently, the elastic relaxation of the spring leads to an increase in the strut diameter. Consequently, our analysis focuses solely on the viscous stretching regime and its corresponding profile near the nozzle, as depicted in Fig. 7b. Equations (18–20) are then employed to determine the elongation viscosity as a function of strain rate (Fig. 7c) for 3 and 4 w/v% alginate solutions. Notably, both solutions demonstrate elongational thinning behavior with increasing strain rate. Moreover, it is evident that the extensional viscosity values for both solutions exhibit no significant difference.

### Impact of cell loading

**Figure 8:**
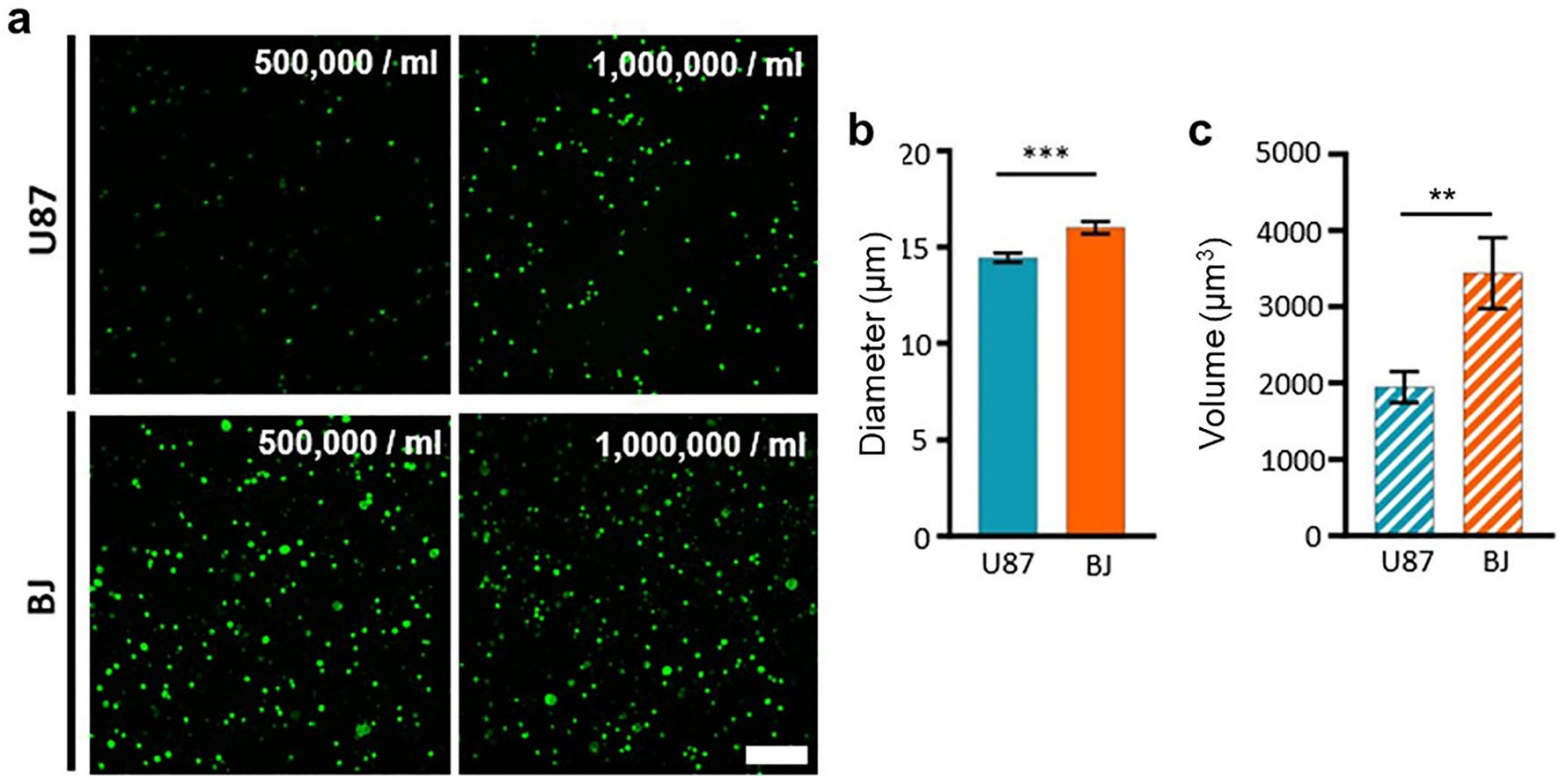
Cells in 4% (w/v) alginate after printing. (**a**) U87 and BJ cells in two different concentrations (left 500.000/ml and 1.000.000/ml) in 4% (w/v) alginate marked in green via immunocytochemical staining for F-Actin. Scale bar represents 200 µm. (**b**) Bar diagram showing mean diameter values of U87 (n = 98) versus BJ (n = 119) cells (students t-test: p = 0.0002 ***). (**c**) Bar diagram comparing the mean volume of U87 cells (n = 30) and BJ cells (n = 34) in 4% w/v alginate (students t-test: p = 0.0067 **).

In 3D bioprinting, cells are typically mixed into the bioink, and instead of pure water, cell culture medium or any other isotonic solution like PBS (Phosphate-Buffered Saline) needs to be used. The isotonic solutions prevent the rupture of cells or shriveling of cells due to osmosis and mimic physiological conditions for the cells. Since the cells behave like a filler in the matrix, they have the potential to influence the rheological behavior and, consequently, the printing performance of the bioink. To analyze this potential influence, cell-loaded alginate solutions were tested. Representative cell loading densities ranging from 150,000 to 1 million cells/ml were employed to simulate conditions relevant to biofabrication. We incorporated U-87 MG (U87) and BJ1-TERT (BJ) cells at different loading densities (150,000/ml, 500,000/ml and 1,000,000/ml) in 4% w/v alginate. The two cell lines differ in size, which consequently allowed potential effects due to cell volume to be included in the investigations. We visualized cells after printing by staining against F-Actin to demonstrate homogeneous distribution of cells within each cell density and hydrogel concentration (Fig. 8a). The cell size was analyzed before printing by measuring cell diameters confirming that U87 cells are significantly smaller (14.45 µm ± 0.26 µm, n = 98) than BJ cells (16.00 µm ± 0.32, n = 119, ***p = 0.0002; (Fig. 8b). We further investigated whether the hydrogel concentration has an impact on cell volume after printing, i.e., if 4% w/v alginate influences either small (U87) or larger (BJ) cells. Cell volume was determined via 3D reconstructions of the F-Actin staining with the help of Imaris Software. Nevertheless, the different cell sizes between small U87 (1949.12 µm3 ± 201.23 µm3, n = 30) and larger BJ cells (3440.68 µm3 ± 466.75 µm3, n = 34, **p = 0.0067) remain as expected (Fig. 8c).

When performing the strut spreading test (“Optical data assessment: Strut-spreading” section) with cell-loaden bio-inks, the first interesting examination was that the utilization of PBS instead of water alters the rheological behavior of the ink. While alginate solutions in water exhibit behavior following the complete wetting model (Eq. 5), PBS-based alginate solutions can be well described by the partial wetting model^27^:

21 $$a(t)={a}_{S}{\left(1-{e}^{- \frac{{4\cdot \gamma }_{L}\cdot 18\cdot {\kappa }^{3}}{\lambda \cdot \eta \cdot {a}_{s}^{7}}\cdot (t+{t}_{0})}\right)}^{1/7}$$

Thus, three adjustable parameters a_s_ (final strut width), B and t_0_ are utilized to fit the data:

22 $$a(t)={a}_{S}{\left(1-{e}^{- B(t+{t}_{0})}\right)}^{1/7}$$

**Figure 9:**
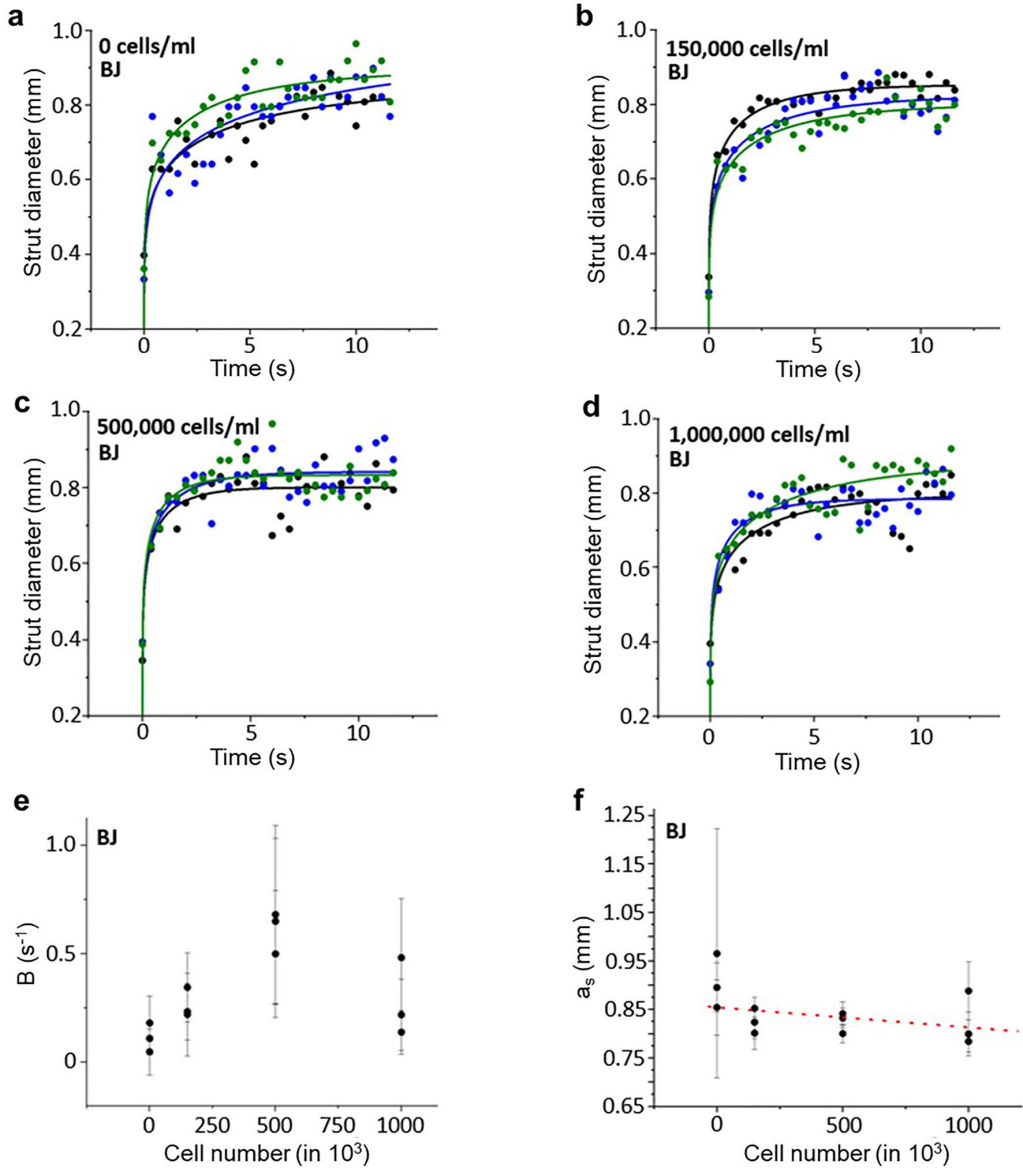
Spreading kinetics of 4% alginate-PBS-solution supplemented with (**a**) 0, (**b**) 150,000, (**c**) 500,000 and (**d**) 1,000,000 BJ cells/ml. The three colors indicate a triplicate of experiments under the same conditions. The solid lines are fits according to theory as described in the text. The parameters B (**e**) and a_s_ (**f**) were by fitting the curves in (**a**–**d**) according to Eq. (22). The corresponding values are plotted against the number of cells incorporated.

The measured curves of a 4% w/v alginate solution with varying cell loading densities were fitted to this model (Figs. 9a–d and S4a–f).

It was once again observed that t_0_ can be effectively set to zero, as its error is comparable to the magnitude of t_0_ itself and generally falls within the range of 1 ms. Comparing the B values (Fig. 9e) and a_s_ values (Fig. 9f), there was no significant impact of cell-densities identified even with the bigger BJ cells, although a_s_ exhibits a marginal decreasing trend (< 3%) with the rise in cell number.

With respect to the initial spreading speed, comparing water and PBS it is useful to consider the tailor expansion up to first order of Eq. (22):

23 $$a(t)={a}_{S} {B}^{1/7}{\left(\frac{t+{t}_{0}}{s}\right)}^{1/7}$$

Considering a negligible ${t}_{0}$, the product ${a}_{S} {B}^{1/7}$ (Eq. 23) and K (Eq. 6) can be compared. Inserting typical values from Fig. 9 (e.g. B = 0.25 and ${a}_{S}$ = 0.83 mm). The corresponding values of 0.688 mm (PBS) are slightly (21%) higher than 0.566 mm (water, see Fig. 5f). From these data, it can be concluded that PBS does increases the spreading speed, while cells have a negligible effect.

## Discussion

The concept of biofabrication, namely the processing of living matter with biomaterials and/or bioactive substances in automated, shaping processes, remains highly complex. For over a decade, scientists from various disciplines have been working on different aspects of biofabrication, including the development of bioinks, process improvement, establishment of cell-stimulating mechanisms, and innovative analytical methods for systematic evaluation of new and existing strategies. Naghieh and Chen recently concluded in a review article that even the concept of printability is inconsistently defined^2^. As a reaction to this problem, initial efforts have been made to establish a more uniform approach within the biofabrication community for testing and documenting new biofabrication approaches with the aim of establishing defined standards. An example is the round-robin study conducted by Garces et al., which involved 15 international groups. The aim was to analyze reproducibility and introduce standards to the bioprinting field in order to accelerate the transition from laboratory practice to production for clinical applications^34^. They concluded that automated image analysis (IA) is a suitable tool for assessing printing process reproducibility, but quantitative comparability in the bioprinting field has not yet been achieved due to lack of standardization in terms of bioprinting equipment. Nevertheless, standardization and normalization in biofabrication, particularly within regenerative medicine, are crucial for advancing future clinical applications. Comprehensive guidelines regarding the classification and terminology of additive manufacturing methods have been established by the American Society for Testing and Materials (ASTM) and the International Organization for Standardization (ISO) through standards such as ASTM F2792 and ISO/ASTM 52900:2021. To specifically address additive manufacturing in the medical field, the United States Food and Drug Administration (FDA) issued "Technical Considerations for Additive Manufactured Medical Devices" (FDA-2016-D-1210), outlining requirements, albeit with less focus on printing hydrogels in a biofabrication approach. Regarding the materials used in fabricating such constructs, ASTM has introduced standards, including ASTM F2150, which evaluates properties for biomaterial scaffolds in regenerative medicine and tissue engineering. This standard involves characterizing the bulk physical, chemical, mechanical, and surface properties of scaffold constructs. Furthermore, optical analytics play a pivotal role in the systematic classification of biomedical scaffolds. ASTM F2603-06 (2020) offers guidance to users in obtaining quantifiable data from tissue scaffold images, evaluating nano- to microstructures using microscopic and tomographic methods. However, these methods are not suitable for online use during printing, emphasizing the need for integration into the process for effective quality management.

The simple imaging setup presented in this work, combined with physical models for the quantitative evaluation of printing behavior, can contribute to a better understanding of material behavior under the specific influence of extrusion pressure, especially when used as a complementary element to existing techniques. In our study, we were able to demonstrate exemplarily how the rheologically determined printing performance of the model system alginate changes depending on the polymer concentration. Additionally, it was demonstrated that the use of PBS instead of water as a solvent has an influence on the flow behavior. In our case, the addition of cells did not lead to a significant change, although such an effect has been demonstrated in other studies with comparable or higher cell loading^35,36^. On the other hand, there are studies that made comparable observations as shown here since at cell densities in the range of 10^6^ cells/ml, only minor effects were observed^37^. Considering the determined cell volume in this study, the cells exhibit a volume fraction ranging from 0.2 to 0.4% vol. They are spherical in shape, resulting in a low surface-to-volume ratio. Additionally, these cells possess a softness characterized by a Young's modulus approximately between 2 and 12 kPa^38–40^. Given these factors, it can be inferred that the printability of the bioink will predominantly depend on its hydrogel properties. Thus, it can be concluded that valuable insights into the potential of the materials used can already be gained through the development of printable bioinks based on existing and newly developed analytical tools, without the need for elaborate printing experiments with cells. Of course, the materials must be simultaneously tested for their biological suitability through cell culture experiments, but at the same time, work can be done more quickly and efficiently on optimizing printing parameters. Though there’s still ongoing demand on standardization, the analytical tools presented in this work might be very useful due to their broad applicability.

## Methods

### Extrusion bio-printing

Alginates (Alginate PH176, Vivapharm, JRS PHARMA GmbH & Co. KG, Germany) at concentrations of 2%, 3%, and 4% (w/v) in H2O were printed using an Inkredible + bioprinter (Cellink Bioprinting AB, Gothenburg, Sweden) equipped with a 25G syringe nozzle (Nordson EFD, Ohio, USA). Extrusion pressures of 16 kPa, 90 kPa, and 130 kPa were respectively applied, calculated to achieve a mass flow of approximately 0.250 mg/s. Similarly, the 5% (w/v) GelMA in PBS (synthesized as described by Loessner et al.43 using ~ 300 g Bloom, Type A, Merck KGaA, Darmstadt, Germany), was printed using a BioX (Cellink Bioprinting AB, Gothenburg, Sweden) with the ink cartridge at 37 °C and a mass flow of 0.250 mg/s. The samples were printed in a strut spreading test meander pattern, utilizing the new imaging setup, onto a glass slide at a printing speed of 5 mm/s. These prints were recorded using a phone camera for at least 60 s to achieve temporal resolution of the strut spreading. The diameter of the strut was then evaluated in triplicate.

### In-gel printing

In-gel printing was conducted by placing a petri dish filled with the support bath onto the Printbase construct setup. If the top of the support bath was not cast perfectly, a slight amount of water would be added to create a smooth top surface. The inks used were 2% (w/v) alginate (Alginate PH176, Vivapharm, JRS PHARMA GmbH & Co. KG, Germany) or 5% (w/v) GelMA (synthesized as described by Loessner et al.^41^ using ~ 300 g Bloom, Type A, Merck KGaA, Darmstadt, Germany), printed into a support bath consisting of 1% (w/v) Xanthan gum (Cosphaderm X34, Cosphatec GmbH, Hamburg, Germany), 0.2% (w/v) hyaluronic acid (80,000–100,000 kDa, FH63427 Biosynth, Staad, Switzerland), supplemented with 7 mM CaCl_2_ for the alginate ink. Printing was conducted using a BioX (Cellink Bioprinting AB, Gothenburg, Sweden), and the process was recorded using a phone camera.

### Strut trajectory

Experimental investigations to evaluate the elongational rheological characteristics of alginate solutions were conducted using the RevoBITs BYTE1 prototype (RevoBITs, Germany, revo-bits.com). This device is equipped with an industry-standard camera, facilitating the capturing of high-resolution images of the strut as necessitated by the experimental protocol. Constant mass flow is ensured by using a direct drive setup for material dispensing. The flow rate is calculated based on the values set for the printing process. Images of the printed strands of 3% and 4% w/v alginate (2% w/v alginate is not printable) dissolved in distilled water are analyzed using ImageJ software 1.54f (National Institutes of Health, MD, USA)^42^. The scale is set to the outer diameter of the 21 G nozzle (Cellink, Boston, MA, USA), 0.82 mm, with an accuracy of two pixels, whereby one pixel corresponds to approximately 0.025 mm. The contours of the upper and lower sides of the strand are detected using the PlugIn "Canny Edge Detector"^43^. Using the OriginLab tool "Average Multiple Curves" (Origin, Version 2021b, OriginLab Corporation, Northampton, MA, USA), the trajectory is obtained. The local diameter is calculated using the nearest neighbor method. According to the BERIT patent^33^, the diameter profile of the strand is fitted with an exponential decay function. The derivative of the pitch angle is determined by applying a linear fit. The local strain rate is computed based on Eqs. (14) and (20). The elongational viscosity is calculated according to Eq. (19). The values of the surface tension are determined using the LCP coefficient method as published by Lee et al.^44^.

### Cell culture

U87 (U-87 MG, ATCC HTB-14, LGC Standards GmbH, Germany) and BJ cells (BJ1-TERT, CVCL_6573) were cultured in Dulbecco's Modified Eagle Medium (DMEM) (41966-029, Gibco, MA, USA) supplemented with 10% FCS (10270-106 Life Technologies, MA, USA) and 100 U mL/ml Penicillin/Streptomycin (15140-122 Life Technologies, MA, USA). The cell lines were split twice per week.

### Measurements of cell diameter

Cells were trypsinized, resuspended in culture medium, and loaded into a cell counting chamber. Brightfield pictures were acquired using an inverted Leica microscope (DM IL LED Fluo, Germany) equipped with a Leica Flexacam C3. Diameters were measured using ImageJ.

### Cell bioprinting

Cells were harvested, counted, aliquoted, pelleted, and the medium was removed to yield the adequate cell concentrations when mixed with 4 ml of 4% alginate in PBS. The cells were dispersed in the ink using a spatula. Subsequently, the cells were bioprinted using an Inkredible + bioprinter (Cellink Bioprinting AB, Gothenburg, Sweden) equipped with a 25G syringe nozzle (Nordson EFD, Ohio, USA), under an extrusion pressure of 130 kPa, calculated to equate to an adequate mass flow of approximately 0.250 mg/s using 4% alginate (Alginate PH176, Vivapharm, JRS PHARMA GmbH & Co. KG, Germany) in PBS as the reference. The cells were printed in the strut spreading test meander pattern onto a glass slide at a printing speed of 5 mm/s, using the Printbase construct, and recorded with a phone camera for at least 60 s to achieve temporal resolution of the strut spreading. The diameter of the strut was then evaluated from triplicates. For cell staining, samples were extruded through the nozzle at the same pressure as the assays were conducted.

### Immunocytochemical staining for the intracellular cytoskeletal protein F-actin

Samples were washed with PBS (pH 7.4) and fixed with 4% PFA/4% sucrose for 10 min at room temperature. After washing with PBS, cells were blocked with 5% BSA/0.1% TritonX-100 in PBS for 30 min at room temperature. They were then incubated for 30 min with ActinGreen™ 488 readyProbes™ reagent (1:50 in blocking solution; R37110 ThermoFisher, Scientific, Germany) and mounted on glass slides with Mowiol 4-88 (81381-50G Sigma Aldrich Chemie, Germany).

### Confocal microscopy and image acquisition

Samples were imaged using an inverted Olympus IX81 microscope equipped with an Olympus FV1000 confocal laser scanning system, a FVD10 SPD spectral detector, and diode lasers 473 nm (Alexa488) (Olympus, Tokyo, Japan). All images shown were acquired using an Olympus UPLSAPO 10× (air, numerical aperture 0.4) or Olympus UPLFLN 40x (oil, numerical aperture: 1.3) objective and were processed using Imaris 7.7.2 (Oxford Instruments, Abingdon, UK). Image stacks at 40× magnification were 3D reconstructed, and the volume of cells was calculated.

### Statistical analysis

GraphPad Prism 8.3.0 (Graphpad Software, San Diego, CA, USA) was used to calculate mean values, standard deviation (SD), standard error of the mean (SEM), and values for statistical significance. Statistical significance was estimated *p < 0.05, **p < 0.01, ***p < 0.001, ****p < 0.0001 using one-wayANOVA or students t-test.

### Supplementary Information

Supplementary Information.

## Data Availability

The datasets generated during and/or analyzed during the current study are available from the corresponding author on reasonable request.
