# Supplementary material for: Advanced optical assessment and modeling of extrusion bioprinting
Source: Sci Rep. 2024 Jun 17;14:13972. doi: 10.1038/s41598-024-64039-y (PMC11183084; doi:10.1038/s41598-024-64039-y)
Supplement: Supplementary file 1 — Supplementary Information. [file 41598_2024_64039_MOESM1_ESM.docx]

**Supplementary Information**


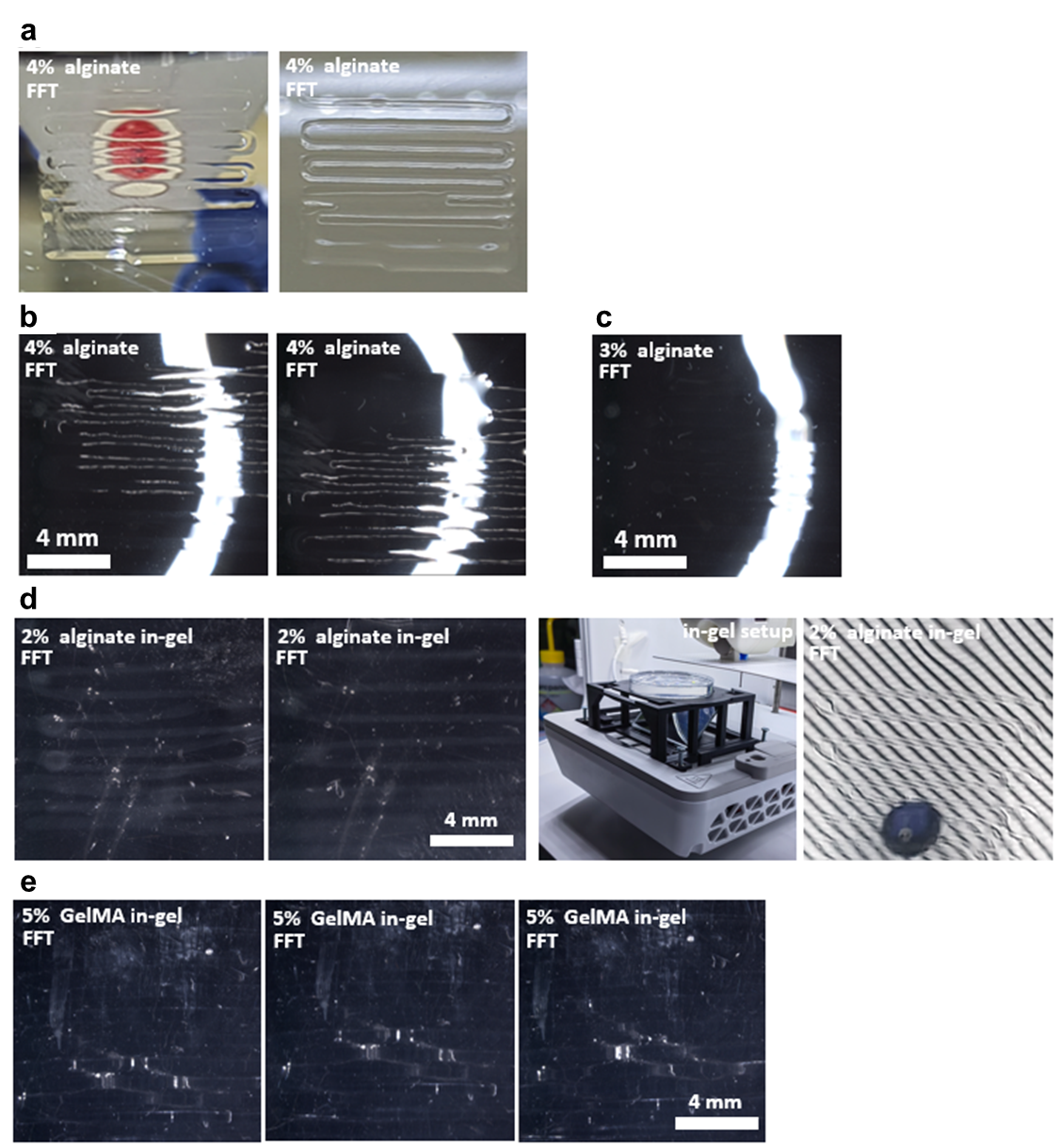


Figure S1: 4% alginate printed onto a glass slide imaged from below without the striped background (a), as well as 4% (b) and 3% (c) viewed under a stereomicroscope. 2% alginate in-gel printed into a xanthan support bath viewed under a stereomicroscope (d), in comparison to the equivalent viewed in the in-gel setup of the print base. 5% GelMA printed into a xanthan support bath viewed under a stereomicroscope (e).


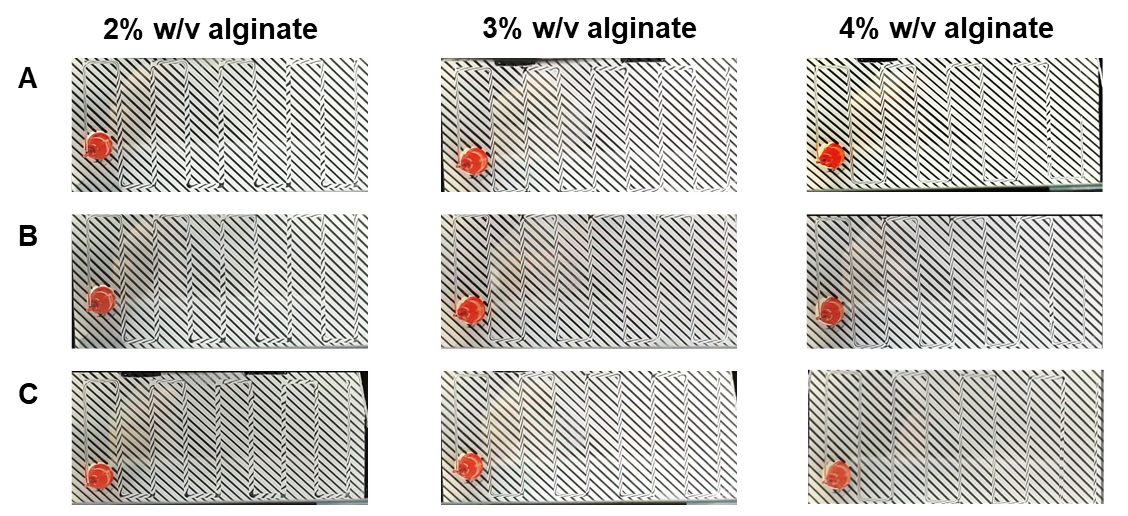


Figure S2: Strut spreading test conducted with 2, 3 and 4% alginate in triplicates indicated by A, B and C.

Table S1: Results from fitting the spreading data of different alginate solutions in triplicates as indicated by A, B and C.

|  |  | **2 %w/v** | **3 %w/v** | **4 %w/v** |
| --- | --- | --- | --- | --- |
| **A** | K (µm) | 736 ± 16 | 522 ± 8 | 531 ± 15 |
|  | $t_{0}$ (ms) | 7 ± 15 | 19 ± 23 | -101 ± 368 |
| **B** | K (µm) | 708 ± 12 | 559 ± 15 | 591 ± 10 |
|  | $t_{0}$ (ms) | 4 ± 7 | -10 ± 421 | 40 ± 45 |
| **C** | K (µm) | 632 ±12 | 592 ± 10 | 577 ± 13 |
|  | $t_{0}$ (ms) | 0.8 ± 2 | 7 ±12 | 22 ± 28 |
| **average** | K (µm) | 692 | 558 | 566 |


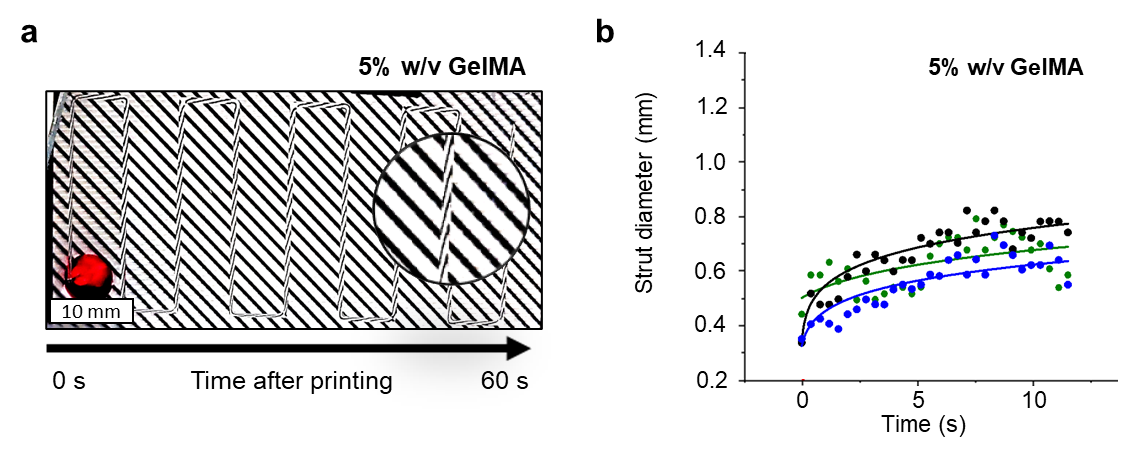


Figure S3: Strut spreading test conducted with 5% w/v GelMa in triplicates indicated by different colors. Exemplary photograph of printed strut from below (a). The K-values of the three independent measurements after fitting (b) with Eq. 6 are 491 µm ± 19 µm, 560 µm ± 7 µm, 461 µm ± 8 µm.


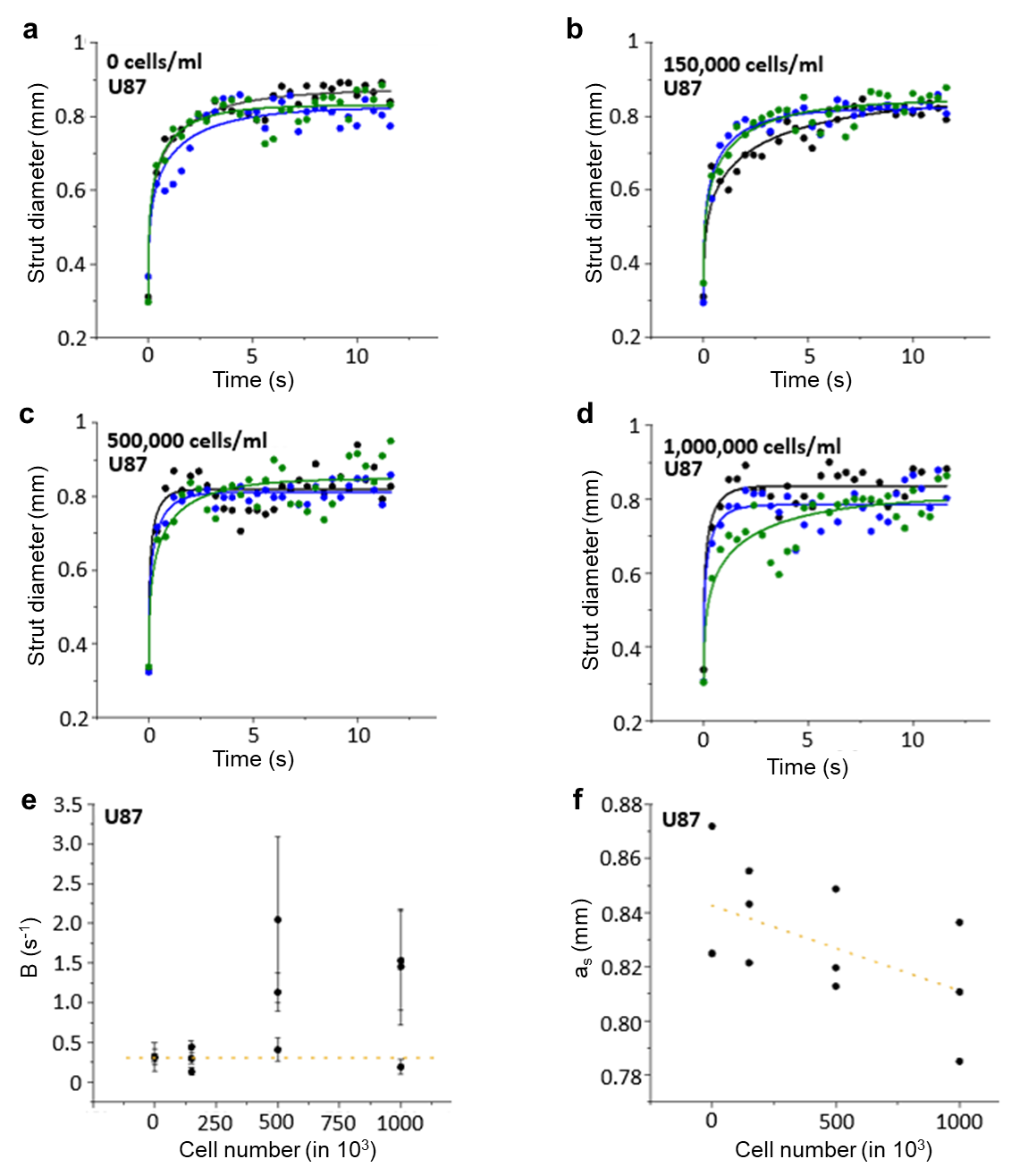


Figure S4: Spreading kinetics of 4% alginate-PBS-solutions supplemented with 0, 150000, 500000, 1000000 cells/ml of U87 cells (a-d). The three colors indicate a triplicate of experiments. The solid lines are fits as described in the text. The parameters B (e) and a_s_ (f) were derived by fitting the curves in a-d according to Eq.22. The corresponding values are plotted against the number of cells incorporated.
